# Conceptualisation, Development, Fabrication and *In Vivo* Validation of a Novel Disintegration Tester for Orally Disintegrating Tablets

**DOI:** 10.1038/s41598-019-48859-x

**Published:** 2019-08-28

**Authors:** Jasdip S. Koner, Ali R. Rajabi-Siahboomi, Shahrzad Missaghi, Daniel Kirby, Yvonne Perrie, Jiteen Ahmed, Afzal R. Mohammed

**Affiliations:** 1 Aston Pharmacy School, Aston University, Birmingham, B4 7ET UK; 2 Colorcon® Inc., Harleysville, PA 19438 USA; 3 Strathclyde Institute of Pharmacy and Biomedical Sciences, University of Strathclyde, Glasgow, G1 1XQ UK

**Keywords:** Biomedical engineering, Characterization and analytical techniques

## Abstract

Disintegration time is the key critical quality attribute for a tablet classed as an Orally Disintegrating Tablet (ODT). The currently accepted *in vitro* testing regimen for ODTs is the standard United States Pharmacopeia (USP) test for disintegration of immediate release tablets, which requires a large volume along with repeated submergence of the dosage form within the disintegration medium. The aim of this study was to develop an *in vivo* relevant ODT disintegration test that mimicked the environment of the oral cavity, including lower volume of disintegration medium, with relevant temperature and humidity that represent the conditions of the mouth. The results showed that the newly developed Aston test was able to differentiate between different ODTs with small disintegration time windows, as well as between immediate release tablets and ODTs. The Aston test provided higher correlations between ODT properties and disintegration time compared to the USP test method and most significantly, resulted in a linear *in vitro*/*in vivo* correlation (IVIVC) (R^2^ value of 0.98) compared with a “hockey stick” profile of the USP test. This study therefore concluded that the newly developed Aston test is an accurate, repeatable, relevant and robust test method for assessing ODT disintegration time which will provide the pharmaceutical industry and regulatory authorities across the world with a pragmatic ODT testing regime.

## Introduction

Orally disintegrating tablets (ODT) are a solid dosage form that disintegrate rapidly upon contact with saliva in the oral cavity. Among the different characterisation methods, disintegration time is one of the most essential attributes to ensure that the ODT disintegrates within the recommended US Food and Drug Agency (FDA) time of 30 seconds or European Pharmacopoeia time of 3 minutes^1^.

**Figure 1:**
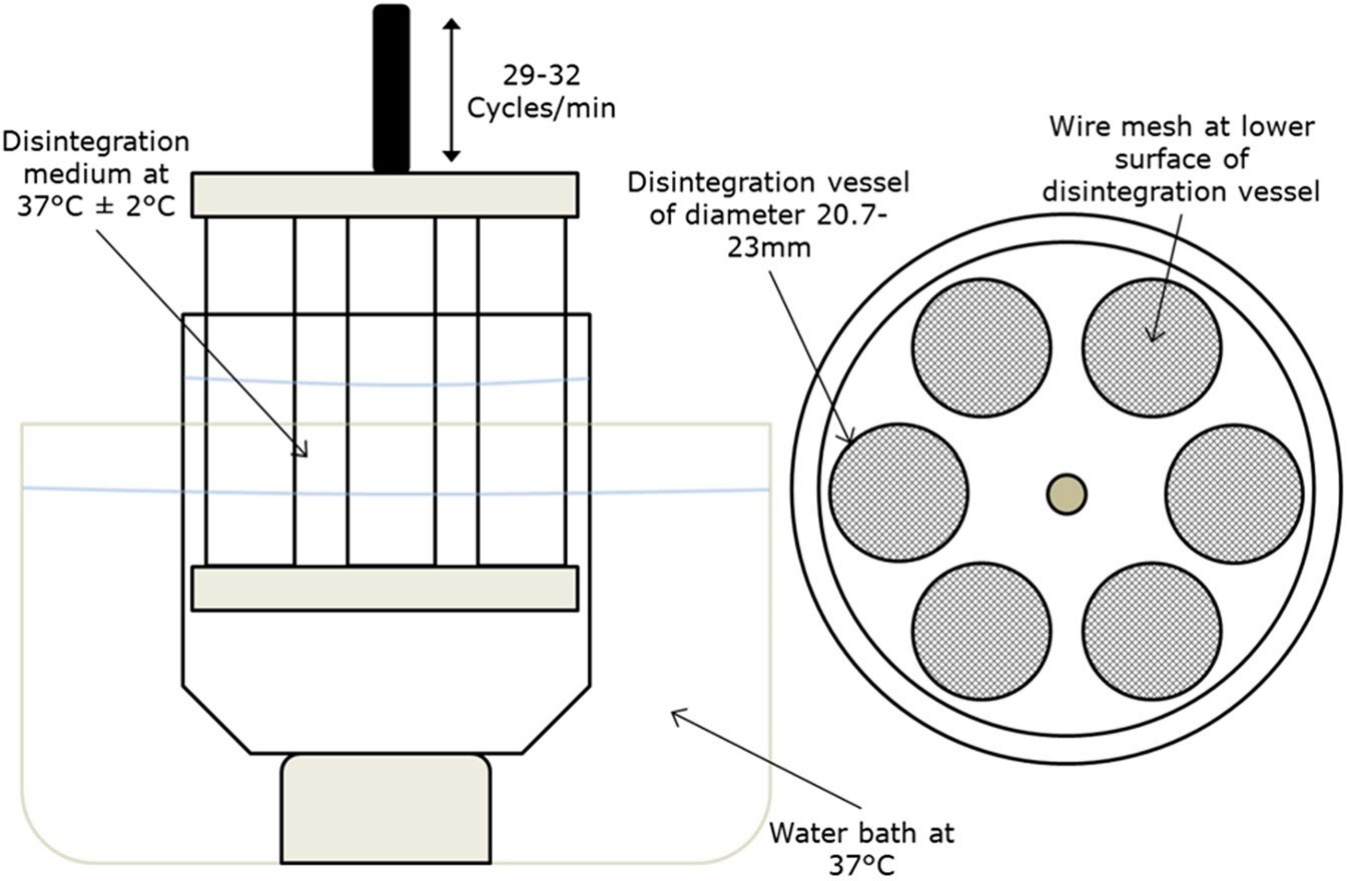
A diagram illustrating a typical set up for the standard USP disintegration test for solid oral dosage forms that is also recommended for ODT disintegration testing. It shows how the basket would typically by placed within the beaker/water bath, and how the dissolution vessels are arranged within the basket.

The currently recommended ODT disintegration test is the United States Pharmacopeia (USP) standard test method used for immediate release solid oral dosage forms, as shown in Fig. 1. This method consists of a basket rack attached to a rod which oscillates vertically within a beaker filled within approximately 800 ml disintegration medium, which is placed beneath the basket assembly and kept at 37 °C. The basket is immersed in the disintegration medium at the frequency of 29–32 cycles per minute through a distance of approximately 53 mm to allow tablet disintegration to take place. The basket is made up of six disintegration vessels with a wire mesh at the bottom (with hole diameters of 1.8–2.2 mm), and disintegration time is measured once all fragments of the oral dosage form have passed through the wire mesh^2^.

However, under *in vivo* conditions, an ODT would be placed on the tongue of the patient and then subsequently disperse/disintegrate through interaction with the saliva present within the oral cavity. As the tablet is placed within the mouth, and the mouth closed; there would be interactions between the ODT and the upper palate in a controlled temperature of around 37 °C^3^, and relative humidity of around 90–95%^4^. These conditions would aid in the disintegration of the ODT, as the high humidity and temperature would promote further moisture uptake into the tablet and the pressing of the tablet against upper palate would further aid in tablet breakdown.

Having considered the *in vivo* conditions, it is clear that the current recommended USP disintegration test method does not bare resemblance to the *in vivo* conditions. The standard test uses a large volume of disintegration medium, and the dosage form disintegrates within the oscillating vessel, which simulates the disintegration of a conventional tablet that is swallowed with water and disintegrates within the gastrointestinal (GI) tract. Also, the time taken for disintegration is measured when the last fragment of the tablet has passed through the wire mesh at the bottom of the vessel, which does not necessarily relate to the time taken for the ODT to disintegrate within the oral cavity and is subsequently ready to swallow.

The USP “Guidance for Industry: Orally Disintegrating Tablets” has stipulated the need for an alternative test for measuring the disintegration time of ODT which bears closer resemblance with *in vivo* conditions^2^. Previous efforts have been made at conducting an *in vivo* relevant/correlating test, with methods ranging from the fabrication of tailored equipment^5–8^, to improvised methods that were developed using currently available lab equipment^9–16^. However, these different tests suffer from various limitations including absence of controlled conditions such as temperature and humidity as well as limited data for demonstrating *in vitro*/*in vivo* correlation (IVIVC).

The aim of the study was to develop an *in vivo* relevant ODT disintegration test method capable of distinguishing marginal differences in the disintegration time of ODTs. This study was designed to develop and conceptualise a new ODT disintegration test which was not only representative of *in vivo* conditions, but also correlated with *in vivo* results. The results from the final design were compared to an *in vivo* ODT disintegration time study.

## Materials and Methods

This study was split in to two stages: 1. Testing of the newly developed Aston disintegration tester (Aston test) and comparing results from tablet properties to the standard USP test method; 2. An *in vivo* study to determine the correlation of *in vivo* results for comparison between Aston test and the standard USP method.

### Materials

Nurofen Meltlets (A marketed ibuprofen 200 mg ODT) and standard immediate release paracetamol tablets were obtained from Reckitt Benckiser (Slough, UK) and Wockhardt (Wrexham, UK) respectively.

Materials for placebo ODT preparation included D-mannitol (Sigma-Aldrich, UK), Microcrystalline Cellulose (MCC) (Avicel PH102, FMC Biopolymer, USA), Partially Pregelatinised Maize Starch (Starch 1500®, Colorcon Inc., USA), crospovidone (Kollidon CL, BASF, Germany), Pearlitol Flash (Roquette, France), sodium stearyl fumarate (Alubra, FMC Biopolymer, USA) and magnesium stearate (Fischer Scientific, UK). Hydroxypropyl methylcellulose (HPMC) (METHOCEL™ K100M, provided by Colorcon Inc., UK) was used to manufacture hydrophilic matrix tablets.

#### Tablet preparation

The placebo ODTs manufactured in this study were composed of 60% w/w D-mannitol, 35% w/w MCC, 4% w/w crospovidone as the main tablet excipients and 1% w/w magnesium stearate as a lubricant. All excipients, except magnesium stearate were blended for 10 mins using a cube blender to achieve a uniform powder blend, and then magnesium stearate added and mixed for a further minute. Individual 500 mg portions of powder were weighed and compressed using a Specac semi-automatic hydraulic press (Slough, UK) equipped with 13 mm flat faced dies. Four compression forces ranging from 75 to 300 MPa were used to more accurately evaluate the sensitivity of the disintegration test.

Extended release matrix tablets were composed of 40% w/w HPMC, 40% w/w MCC, 19% w/w D-mannitol and 1% w/w magnesium stearate. All the excipients were blended as above. The powders were individually weighed to 500 mg and tableted using the Specac semi-automatic hydraulic press (Slough, UK), with the same 13 mm flat-faced dies as above, followed by compression at 225 MPa.

#### Validation of disintegration time using Aston test

Two commercially available tablets, an ODT formulation, Nurofen Meltlet and a standard release paracetamol were used as model products. Five sets of placebo tablets were manufactured to test the time sensitivity within the Aston test; four ODTs compacted at different compression forces, to give varying hardness and disintegration time profiles, and an extended release matrix tablet, to give a control that should not disintegrate within the Aston/USP test.

#### Aston disintegration test

**Figure 2:**
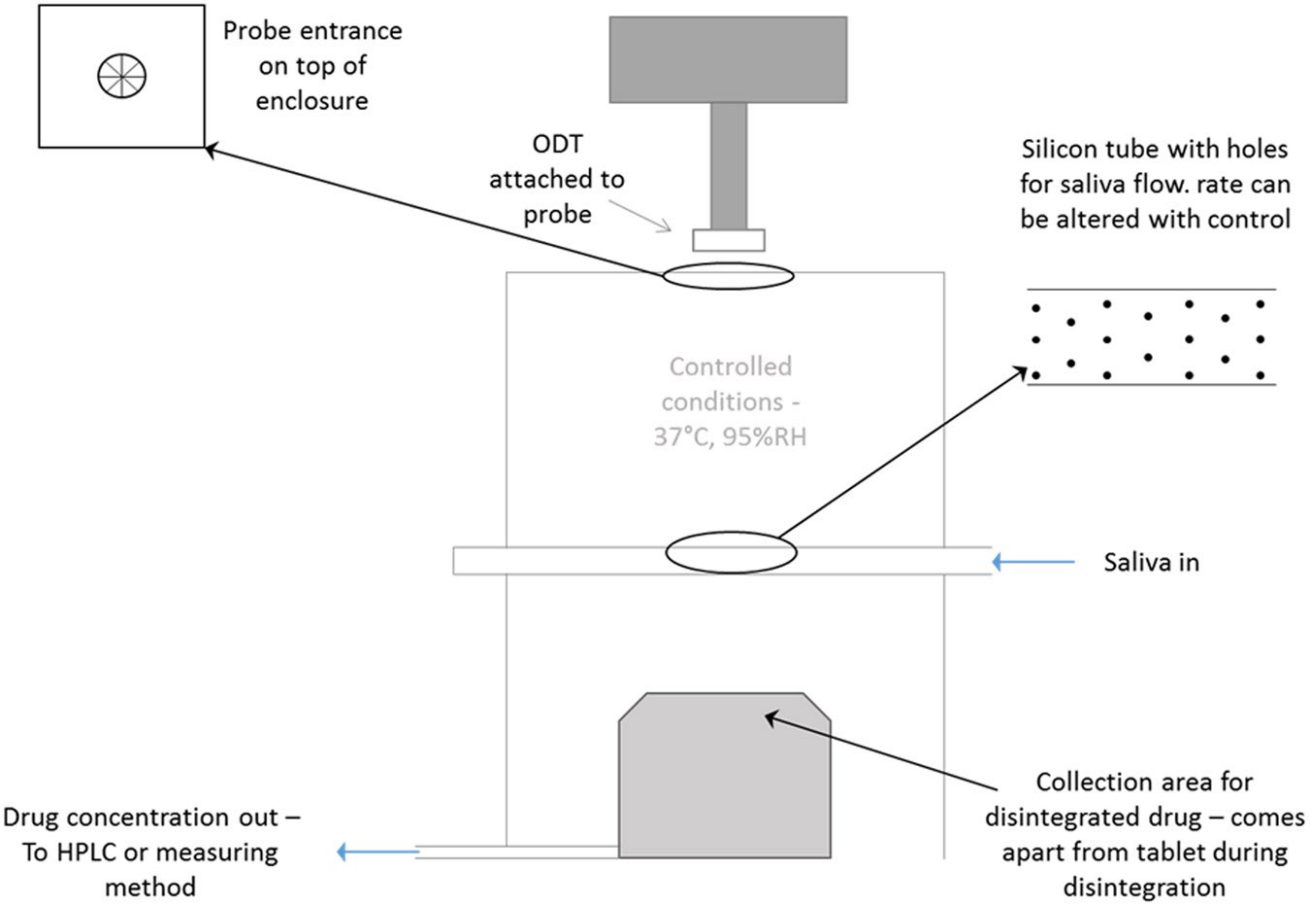
A schematic diagram showing the proposed design of the disintegration tester specific for ODTs, comprising of conditions representative of the oral cavity, including temperature/humidity, disintegration medium flow rate and applied pressure on the tablet. An area for collection of the disintegrating fragments from the tablet could also be added to assess drug leakage/absorption in the mouth.

The newly developed Aston test was fabricated according to the drawing illustrated in Fig. 2, which was designed to mimic conditions encountered in the oral cavity. The test housing was placed on top of a hot plate, set to an optimised temperature to achieve 37 ± 1 °C within the compartment, similar to *in vivo* conditions^3^. The test housing contained potassium chloride which was used to form a saturated salt solution to provide relative humidity of approximately 93 ± 3% RH in the enclosed container, similar to those conditions encountered in the oral cavity^17^. The disintegration bed was a slightly flattened silicone pipe with 4 mm holes to allow water or simulated saliva to flow over the surface of the pipe. This flow of media would interact with the tablet and lead to subsequent disintegration within the simulated *in vivo* conditions. The flow rate of simulated saliva was set at 10 ml/min to form a thin film of liquid over the silicone pipe.

The disintegration compartment was placed under the probe of a texture analyser (Brookfield Engineering’s CT3 Texture Analyser, Harlow, UK), set at speed of 2 mm/s. Once the tablet came into contact with the disintegration bed, the probe was set to apply a fixed 50 g weight for a set amount of time^12,13^. A plot of distance vs time was then generated from which disintegration time was calculated. The test was repeated on eight tablets and data was presented as mean ± standard deviation (SD).

#### USP disintegration test

The standard USP disintegration method was also used to assess disintegration time of the tablets to allow a comparison to the Aston test^18^. A Copley ZT41 disintegration apparatus (Nottingham, UK) was used, with a single tablet being tested at one time for accuracy. Each tablet was placed in the vessel (without a disk) and oscillated at 30 cycles per minute. A dissolution medium of 800 ml distilled water was maintained at 37 °C, and disintegration time measured when all of the fragments of tablet had passed through the mesh at the bottom of the vessel. All readings were taken in triplicate and represented as mean ± SD.

#### Hardness

Tablet hardness was measured using a Copley TBF 100 Hardness tester (Nottingham, UK). Tensile strength (σ) was then calculated using the equation:

1 $$\sigma =\frac{2\,H}{\pi \,D\,T}$$

where H is the hardness, D is the diameter and T is the tablet thickness. All readings were taken in triplicate and displayed as mean ± SD.

#### Porosity

Porosity of the tablets was assessed using a Quantachrome Helium Multipycnometer (Florida, USA). Diameter and thickness of the ODTs were measured using a digital calliper, and the weight of individual tablets was determined using an electronic balance. The bulk volume (V_B_) and bulk density (ρ_bulk_) of the tablets were then calculated using the following equations:

2 $${V}_{B}=\frac{\pi \,{R}^{2}}{T}$$

3 $${\rho }_{bulk}=\frac{Tablet\,weight}{{V}_{B}}$$

The true volume (V_t_) of the tablet was calculated using the pycnometer, which applies the theory of gas displacement allowing the porous nature of the tablet to be assessed. The true volume was calculated using the equation:

4 $${V}_{t}={V}_{c}-{V}_{r}(\frac{P1}{P2}-1)$$

where V_C_ is the volume of the sample cell, V_r_ is the volume of the reference cell, P1 and P2 are the atmospheric pressure and pressure change during the measurement respectively. The true volume was then used to calculate true density in the equation:

5 $${\rho }_{true}=\frac{Tablet\,weight}{{V}_{t}}$$

The final step to calculate porosity (ε) of the ODT used the following equation:

6 $$\varepsilon =1-\frac{{\rho }_{bulk}}{{\rho }_{true}}$$

### *In Vivo*/*In Vitro* correlation (IVIVC)

#### *In vivo* disintegration time assessment

*In vivo* disintegration time was investigated using nine different tablets across 35 healthy human volunteers. The study design is detailed below.

#### Study design

The study was subject to ethics approval from Aston University Ethics Committee and gained a favourable opinion letter for commencement of the study based on the Research Protocol, Patient Information Sheet and Patient Consent form. The lead investigator, Jasdip Koner, was also certified in Good Clinical Practice through the National Health Service (UK). The study was performed in accordance with the relevant guidelines and informed consent was obtained from all participants.

The study was designed as a single blind study whereby participants were not aware of the tablet type they were taking. Each participant was assigned a participant number according to their chosen seat in the study room, which was used by the researchers to assign which tablets were taken. Participants were required to take a total of six tablets per scheduled study, the participant was not privy to formulation composition. Participants followed a set of instructions laid out by the research team. The tablets were taken in the defined order set out by the researchers, and times recorded from when the tablet entered the oral cavity to when the participant felt the tablet had disintegrated. Participants were briefed on when the endpoint of disintegration should be/feel like. Prior to taking the tablet, participants had to rinse the oral cavity, as well as rinsing at the end of each tablet disintegration and before the start of each test. A wait time of 1–2 minutes was advised to allow oral conditions to return to the resting state before moving on to taking the next tablet. Participants recorded their own disintegration time using stop watches. The study involved no swallowing of the tablet and the participants were informed that all residue was to be removed from the oral cavity. Once all studies had been completed, results were collated. No patient demographic data was collected and there was also no patient identifiable data, as participants chose their own number/seat at the study.

#### Participant recruitment

A total of 35 healthy participants took part in the i*n vivo* disintegration time study based on the selection criteria outlined in the Research Protocol, Patient Information Sheet and Patient Consent Form. Participants were required to commit up to 60 mins for the study. Participants were recruited from Aston University staff and were subject to inclusion and exclusion criteria to determine eligibility for the study. The volunteers were sent participant information sheets and consent forms prior to the study and allowed to make their own informed decision on study participation.

#### Manufacture of tablets

A total of five different powder blends were prepared, with four blends compressed at two different compression forces and the fifth blend compressed using only one force (Table 1), giving a total of nine different batches of tablets. Tablets were composed of Pearlitol Flash (Roquette, Lestrem, France), Starch 1500 (Colorcon Inc., Harleysville, USA) and magnesium stearate (Fischer Scientific, Loughborough, UK) (Table 1). Excipient concentration and compression force was varied to obtain tablets of differing disintegration times, to allow for a wide range of times to be compared between the *in vivo* disintegration times and the times obtained for the *in vitro* methods.

**Table 1:** Tablet formulation details for the 9 batches of tablets utilised in the *in vivo* disintegration study.

Formulation	Batch	Pearlitol Flash (%)	Starch 1500 (%)	Magnesium Stearate (%)	Compression Force (KN)
Powder Blend 1	B1	99.5	—	0.5	4
	B2				9.5
Powder Blend 2	B3	89	10	1	9.5
	B4				22
Powder Blend 3	B5	79	20	1	20
	B5				46
Powder Blend 4	B7	49.5	49.5	1	26
	B8				57
Powder Blend 5	B9	24.5	74.5	1	32

Each powder blend, except powder blend 5, were compressed at two different compression forces to provide tablets of different disintegration/hardness profiles.

#### Data analysis

Each participant was required to take a total of six tablets, this comprised of two different tablet batches in triplicate. Each individual tablet batch was assessed 21 times; a participant took a particular tablet batch in triplicate, giving three readings, and a particular tablet batch was tested across seven different participants giving a total of 21 single readings per tablet batch. This approach was taken to gather not only inter-person variability but also intra-person variability, whilst also providing a very robust mean value. Data was presented as mean ± SD.

#### *In Vitro* tablet disintegration time assessment

The tablet formulations outlined in Table 1 were also tested in the standard USP test and Aston test. Each tablet batch was repeated in triplicate and data presented as mean ± SD.

### Statistical analysis

One-way and two-way analysis of variance (ANOVA) followed by Tukey’s and Sidak’s multiple comparison post-hoc tests respectively, were performed in this study, using GraphPad Prism 6 software (California, USA). For statistical significance a p-value < 0.05 was used, and all data was presented as mean ± SD.

## Results and Discussion

### Validation of disintegration using newly developed aston disintegration tester

Although several attempts have been made to design a test that is more specific to ODTs^5–7,9,10,12–16,19–26^, no test has reached approval specifically for ODT disintegration testing. The Aston test was conceptualised and developed as there is currently no validated test that broadly mimics the oral *in vivo* conditions encountered by an ODT during disintegration.

A systematic approach was taken to formulate ODTs with varying disintegration times by changing the compression force from 75 to 300 MPa. Hardness and porosity values for all tablets are reported in Table 2. It was noted that as the compression force increased, tablet hardness and disintegration time of the ODTs increased, as expected. This was due to the particles in the compact interacting more closely and being bound tightly during the compression cycle, owing to the high content of MCC as a plastically deforming excipient^27^.

**Table 2:** Tablet properties for all formulations tested in this study.

Formulation	Aston Test Disintegration Time (s)	USP Disintegration Time (s)	Hardness (N)	Tensile strength (N/mm^2^)	Porosity
75 MPa	22.51 ± 3.2	13.83 ± 0.25	58.67 ± 2.95	0.899 ± 0.076	0.399 ± 0.005
150 MPa	28.11 ± 2.5	14.47 ± 1.42	121.37 ± 7.60	2.120 ± 0.133	0.320 ± 0.010
225 MPa	35.27 ± 2.07	22.70 ± 2.00	154.87 ± 3.80	2.766 ± 0.062	0.314 ± 0.10
300 MPa	42.15 ± 2.07	28.60 ± 1.31	177.33 ± 6.12	3.267 ± 0.109	0.292 ± 0.011
Nurofen Meltlet	38.77 ± 3.96	15.80 ± 0.10	44.37 ± 2.41	0.447 ± 0.024	0.368 ± 0.004
Matrix	180.00 ± 0.00*	1800.00 ± 0.00*	173.70 ± 20.87	3.290 ± 0.575	0.300 ± 0.022
Paracetamol	180.00 ± 0.00*	97.60 ± 5.99	144.80 ± 10.20	1.875 ± 0.137	0.270 ± 0.013

All data is presented as mean ± SD, with data marked with an asterisk (*) showing the maximum time for the disintegration test, and therefore shows this tablet had not disintegrated within the stated time and was still left as a solid mass.

Disintegration times for the seven different formulations are shown in Table 2. The results showed that Aston test was sensitive to detect small differences in disintegration time (as little as 7 seconds). An important investigation in this study was to determine whether this test would be able to differentiate between an ODT and a standard immediate release/extended release tablet that would not usually disintegrate within the oral cavity. Table 2 shows that the matrix tablet and the standard release paracetamol tablet had not disintegrated within the 180 s limit set for the Aston test. Upon visual evaluation, the matrix tablet was almost fully intact with a thin hydrated layer at the bottom of the tablet facing the media. Similarly, the paracetamol tablet remained intact, however a slightly larger proportion of the tablet had eroded and broken off the main structure.

**Figure 3:**
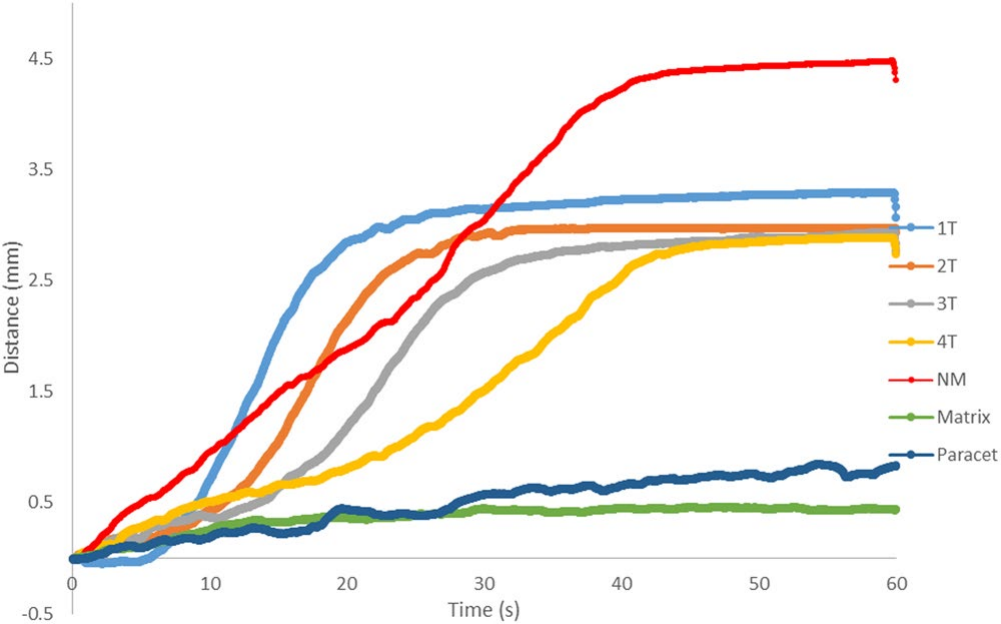
Disintegration profiles (distance vs time plots) of the seven formulations tested within the Aston test in this study, showing marked differences between disintegration pathways for ODTs and standard/controlled release formulations, and statistical significance observed (n = 8, p < 0.05).

Figure 3 illustrates the differences between the disintegration pathways of the evaluated ODTs and the non-disintegrating tablets (i.e. matrix-controlled release system and the standard release paracetamol tablets). It was deduced that all the ODTs followed a similar pathway, an initial small flat region, where water and humidity started to ingress into the tablet to break down the structure, as the tablet was not yet fully wet and still retained some mechanical strength; This was followed by a sharp increase in distance as the tablet was breaking down and falling apart from the main structure and away from the disintegration bed. This caused the probe to press further into the structure, given that there was very little resistance from the soft wet tablet. The final stage was when the probe had pushed through the ODT and on to the surface of the disintegration bed, whereby the plot came to a plateau as the probe reached its maximum force of 50 g. The plots also showed that there was a difference between the disintegration times for the ODTs manufactured at different compression forces (p < 0.05). The disintegration pathway for the ODTs showed that there was a difference in thickness of the tablets compressed at different forces, as the final distance for the tablets compressed at higher forces was smaller than those compressed at lower forces. This supported the theory that the test was sensitive enough to identify minor differences in disintegration time due to differences in compression force, mechanical strength and porosity.

However, for the extended release matrix tablet and the standard immediate release paracetamol tablet it was noted that the disintegration pathway was very different, with neither tablet undergoing significant disintegration. Both of these tablets are formulated to be swallowed whole and enter the GI tract, whereby the tablet would subsequently disintegrate in a large volume of fluid over longer duration beyond 180 seconds. The paracetamol tablet was an immediate release dosage form, so it would disintegrate in the stomach in a relatively short time, whereas the matrix tablet was an extended release system that would slowly swell and erode layer by layer to provide sustained release of drug. The disintegration profile for both was similar at the start, where the water from underneath the tablet had come into contact with the tablet and the tablet interacted with the humidity in the chamber, providing a small increase in distance. However, after this initial movement, it was observed that the probe moved a very short distance (<0.5 mm). This was because the tablet remained rigid, as the media and humidity could not ingress into the tablet core and cause a breakdown of the structure, resulting in the probe applying its maximum set weight of 50 g throughout most of the test.

The comparison between ODTs and standard/extended release tablets was essential as the results indicated that the tester was able to clearly distinguish between different types of tablets. It also demonstrated that an ODT would disintegrate when tested but the other types of tablets remained intact, as they were manufactured to disintegrate or dissolve in larger volumes of fluid as opposed to the oral cavity.

### Comparison to the USP test

**Figure 4:**
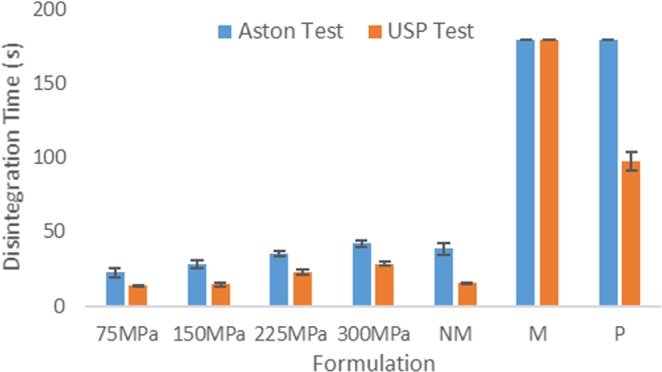
A graph comparing disintegration times for the Aston disintegration test and the standard USP test, showing that for ODTs in the USP test provide lower disintegration times than the Aston test. Both values for the matrix tablet and the disintegration time for the paracetamol both reach the maximum time of 180 s in the Aston test and 1800 s in the USP test for the matrix tablet only; Values displayed on the graph have been set at 180 s to represent the maximum. Data is presented as mean ± SD (n = 8 for Aston test and n = 3 for the USP test, p < 0.0001 for all data (except matrix tablet) when the Aston test is compared to the USP test, indicating that results are significantly different between the two tests). (Abbreviations: NM – Nurofen Meltlet; M – Matrix tablet; P – Standard release paracetamol).

It was established that the newly developed disintegration tester was able to successfully determine different disintegration times and profiles between ODTs and standard release/extended release matrix tablets, whilst also providing different disintegration time between ODTs manufactured at different compression forces. The next important investigation was to analyse how the test compared to the standard USP disintegration test, especially for those ODTs manufactured in the laboratory. Figure 4 shows a comparative graph of the disintegration time for the Aston test and the USP test for all evaluated tablets.

The disintegration times for the four ODTs and the Nurofen Meltlets® appeared to be lower when tested in the USP test rather than the Aston test. This was expected as the conditions in the USP test, submerging the tablet in 800 ml of disintegration medium and oscillating it through the medium promoted rapid disintegration of the dosage form. This, alongside the mechanical agitation during the oscillations, resulted in rapid disintegration of the dosage form, compared to the conditions that would be encountered in the oral cavity^6,10,13,26,28^. In comparison, the Aston test consists of controlled flow of disintegration fluid whilst maintaining relative humidity in the disintegration chamber and was more representative of *in vivo* conditions compared to the conventional USP test.

It was observed that both the standard release paracetamol and matrix tablets experienced very little disintegration during the Aston test, with results represented as the maximum set time of 180 s in Fig. 4. The matrix tablet also experienced very little disintegration during the standard USP test and remained intact at 30 mins. However, the standard release paracetamol tablet clearly disintegrated during the standard USP test at around 97 s. Upon visual analysis, and the analysis of the disintegration profile (Fig. 3) it was observed that the paracetamol tablet underwent very little disintegration during the Aston test, due to the small volume of disintegration fluid and controlled relative humidity.

**Figure 5:**
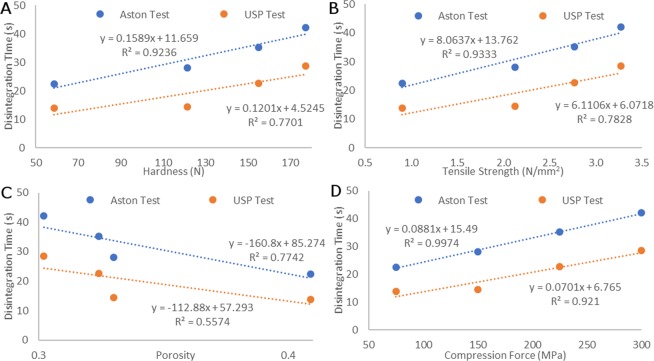
**A**) A graph comparing the correlation between hardness and disintegration time data for both the Aston test and the standard USP test, with results indicating a better correlation with the Aston test; (**B**) A graph comparing the correlation between tensile strength and disintegration time data for both the Aston test and the standard USP test, with results indicating a better correlation with the Aston test; (**C**) A graph comparing the correlation between porosity and disintegration time data for both the Aston test and the standard USP test, with results indicating a better correlation with the Aston test and (**D**) - A graph comparing the correlation between compression force of the tablet press and disintegration time data for both the Aston test and the standard USP test, with results indicating a better correlation with the Aston test.

Figure 5 shows the correlations for hardness, tensile strength, porosity and compression force against disintegration time, for both the Aston test and the USP test. For this analysis, the standard release paracetamol tablets and matrix tablets were excluded as the comparison was ODT specific. All results indicated that the Aston test provided higher correlations, with the R2 (correlation factor) values for hardness, tensile strength, porosity and compression force being 0.92, 0.93, 0.77 and 0.99 respectively, compared to the lower respective values of 0.78, 0.77, 0.56 and 0.92 obtained using the USP test. The correlation between hardness and tensile strength values (Fig. 5(A,B) respectively) was very high for the Aston test, with the USP test correlation falling far below this. This showed that in terms of disintegration time, mechanical strength played an integral part, correlating almost linearly with disintegration time in the case of the Aston test under simulated *in vivo* conditions. In terms of the porosity of the tablet (Fig. 5C), the Aston test demonstrated a strong positive correlation, with an R2 value of 0.77, providing evidence that porosity also played a key role in the disintegration time of ODTs. On the other hand, the correlation for the USP test indicated a weaker correlation between the disintegration time and mechanical strength/porosity, suggesting that disintegration time didn’t strongly relate to these important properties of ODTs in the USP test.

Accordingly, based on the results gathered in this section, the Aston test proved to be a relevant method for measuring disintegration time for ODTs, as it provided more representative conditions to those encountered i*n vivo* and also provided much stronger correlations to tablet properties compared to the standard USP test.

### *In vivo*/*In vitro* correlation

**Figure 6:**
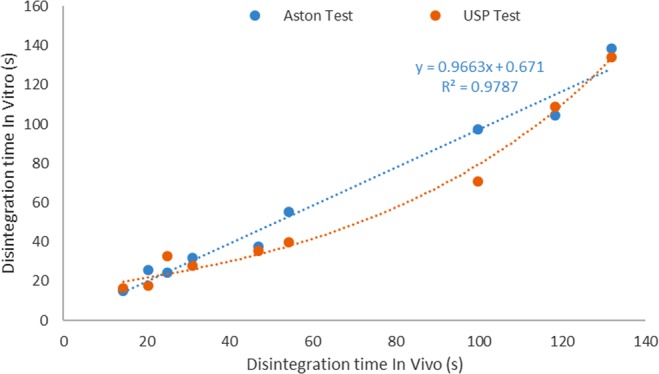
A graph showing the IVIVC of the newly developed Aston disintegration test compared to the standard USP disintegration test. Results show that the Aston test has a linear correlation for the *in vivo in vitro* disintegration times whereas the USP tester appears to show a curved data set therefore indicating a direct correlation is not observable with the USP test.

The *in vivo* disintegration times for all batches were compared and correlated with results from the Aston test, as well as with the USP test (Fig. 6). The collection of 21 individual results per tablet for the *in vivo* study provided robust data for wide representation of disintegration times. To supplement this, results from seven participants were taken to calculate the mean for each tablet thereby ensuring inter- and intra-person variability was captured in data analyses. The tablets were manufactured varying compression forces to provide disintegration time profile from 10 to 150 s to enable correlation of results accepted by the EU regulations (180 s)^1^ or the regulations recommended by the FDA (30 s)^2^.

Figure 6 shows the IVIVC for tablets spanning both the acceptable range for the EU and FDA regulation. Results indicated that the Aston test showed high linear correlation (R2 value 0.98) with the *in vivo* results. It was also observed that the Aston test was highly sensitive, whereby 1 second increase in *in vivo* disintegration time correlated with proportionate increase with Aston test. However, in comparison, the results obtained for the USP tester were not linear (with a curved dataset), indicating that the USP test was neither sensitive nor provided direct representation of the disintegration time for ODTs *in vivo*. Further analysis of the data, where ODTs with disintegration time meeting USP standards (<30 s) showed that the Aston test provided strong linear correlation, with an R2 value of 0.87, as opposed to USP test which had a very weak linear correlation of 0.61.

## Conclusion

The objective of this study was to develop and assess a novel disintegration test for ODTs which was constructed to simulate *in vivo* conditions the tablet would encounter during administration. Results from this study demonstrated that the Aston test was sensitive and capable of distinguishing subtle differences in ODT disintegration time profile. A robust *in vivo* study design confirmed that the Aston test simulated the conditions (saliva volume, temperature, humidity) encountered in the oral cavity and provided a practical test representative of *in vivo* test data. The Aston test conclusively demonstrated that the novel method is accurate, precise, reproducible and linear thereby offering a pragmatic alternative to *in vivo* human subject testing. Standardisation of ODT disintegration test using the Aston test will ensure consistency in disintegration time data, which is a unique attribute for an ODT.
